# Lessons learned from 10 years of experience with minimally invasive cardiac surgery

**DOI:** 10.3389/fcvm.2022.1053572

**Published:** 2023-01-09

**Authors:** Ali El-Sayed Ahmad, Saad Salamate, Farhad Bakhtiary

**Affiliations:** ^1^Department of Cardiac Surgery, Heart Center Siegburg, Siegburg, Germany; ^2^Department of Cardiac Surgery, University Hospital Bonn, Bonn, Germany

**Keywords:** minimally invasive surgery, cardiac surgery, lessons learned, aortic valve, mitral valve

## Abstract

Since its inception more than a quarter of a century ago, minimally invasive cardiac surgery has attracted the increasing interest of cardiac surgeons worldwide. The need to surgically treat patients with smaller and better-tolerated incisions coupled with high-quality clinical outcomes, particularly in structural heart disease, has become imperative to keep pace with the evolution of transcatheter valve implantation. We have learned numerous lessons from our longstanding experience in this field of surgical care, especially in terms of endoscopic access *via* mini-thoracotomy. To improve the safety and efficacy of this minimally invasive endoscopic access, this study summarizes and highlights the lessons we have learned, acting as a template for newly established cardiac surgeons in minimally invasive techniques.

## Introduction

The term “minimally invasive cardiac surgery (MICS)” covers a vast field of procedures, including valve surgeries, coronary artery bypass graft surgery (CABG), and intracardiac tumor resections, and has shown success in terms of safety and efficacy compared to conventional sternotomy (CS) (1). MICS techniques allow heart operations to be performed and enable access to the relevant anatomical structures through substantially smaller incisions. These patient-friendly techniques in turn help to avoid the excessive dissection of surrounding tissues and even circumvent the need for a cardiopulmonary bypass (CPB), thereby leading to a faster post-operative recovery period and a cosmetically superior result for the patient (2). Although MICS approaches provide indisputable benefits to the patient, most studies report a correspondingly longer time for extracorporeal circulation and cardiac arrest with the minimally invasive approach. However, MICS benefits both from the utilization of video-thoracoscopic assistance and from advancements in CPB techniques to offer advantages such as a reduction in cross-clamp times, CPB times, and ventilator support, as well as shorter intensive care and total hospital time (3, 4).

Minimally invasive cardiac surgery approaches mainly include the upper and the lower mini-sternotomy, parasternal approach as well as the right and the left mini-thoracotomy to perform isolated or multiple valve surgery, CABG, intracardiac tumor resections, or atrium septum defect closure, considering the peculiarities of each technique for each procedure. In the last few years, the use of endoscopic minimally invasive access *via* mini-thoracotomy to reach different cardiac structures has gained popularity among surgeons who perform minimally invasive cardiac surgery. The steep learning curve and technical difficulties of the different procedural steps such as CPB cannulation, myocardial protection, and deairing maneuvers discourage many surgeons from including these minimally invasive procedures in their routine surgical practice (5).

This study summarizes the lessons learned from our decade-long experiences using different approaches to MICS, with an emphasis on minimally invasive endoscopic mini-thoracotomy to encourage cardiac surgeons to adopt this technique for safety and feasibility.

## Lesson 1: Patient selection

In our opinion, the final decision on the operative strategy for each patient requires that cardiac surgery be performed on an individual-to-individual basis during a pre-operative medical staff meeting by taking into consideration the pre-operative demographic data of the patient such as age, comorbidities, vascular status, and EuroSCORE II (European System for Cardiac Operative Risk Evaluation II). The choice of the surgical technique is the net result of the application of an internal policy recommendation, which is exactly tailored to meet the requirements of an individual patient. An increased EuroSCORE and the age of the patient alone are not contraindications for endoscopic MICS. Elderly patients benefit even from the slightest advantages of endoscopic MICS by decreasing surgical trauma and perioperative pain, blood transfusions, hospital and intensive care unit (ICU) length of stay, ventilation time, wound infections, cost of hospitalization, and rehabilitation when compared to the CS. Moreover, for the young patient group, these techniques improve the cosmetic, quality of life, and patient satisfaction with an earlier return to normal activities (3, 4, 6). Even patients with difficult anatomical conditions, such as pectus excavatum or dextrocardia by situs inversus (DSI), are suitable for an endoscopic MICS when performed at experienced MICS centers. We published the first report of an endoscopic aortic valve replacement (AVR) through left anterior mini-thoracotomy in a patient with DSI. The recognition of anatomical abnormalities through a careful evaluation of the pre-operative diagnostics and the rearrangement of the operation theater equipment in a mirror-image fashion by adapting the surgical technique to the reversed anatomy are fundamental to the success of this concept (7).

Several structural pathologies of the heart including extensive endocarditis or severe calcification of the mitral valve (MV) annulus or severe calcification of the abdominal and/or iliac aorta are major limitations of this technique (Table 1). Therefore, computer tomography angiography (CTA) of the aorta and the arterial vascular system remains a very important pre-operative diagnostic tool for deciding a patient's eligibility for an endoscopic MICS procedure.

**Table 1:** Contraindications for endoscopic minimally invasive cardiac surgery.

**Extensive endocarditis**
Several calcifications of the aortic or mitral valve annulus
Several calcifications of the thoracal and/or abdominal aorta
Hostile aortic root
Severe peripheral artery disease
Severe adhesions of the lung
Extreme left deviated heart axis

## Lesson 2: Surgical equipment

**Figure 1:**
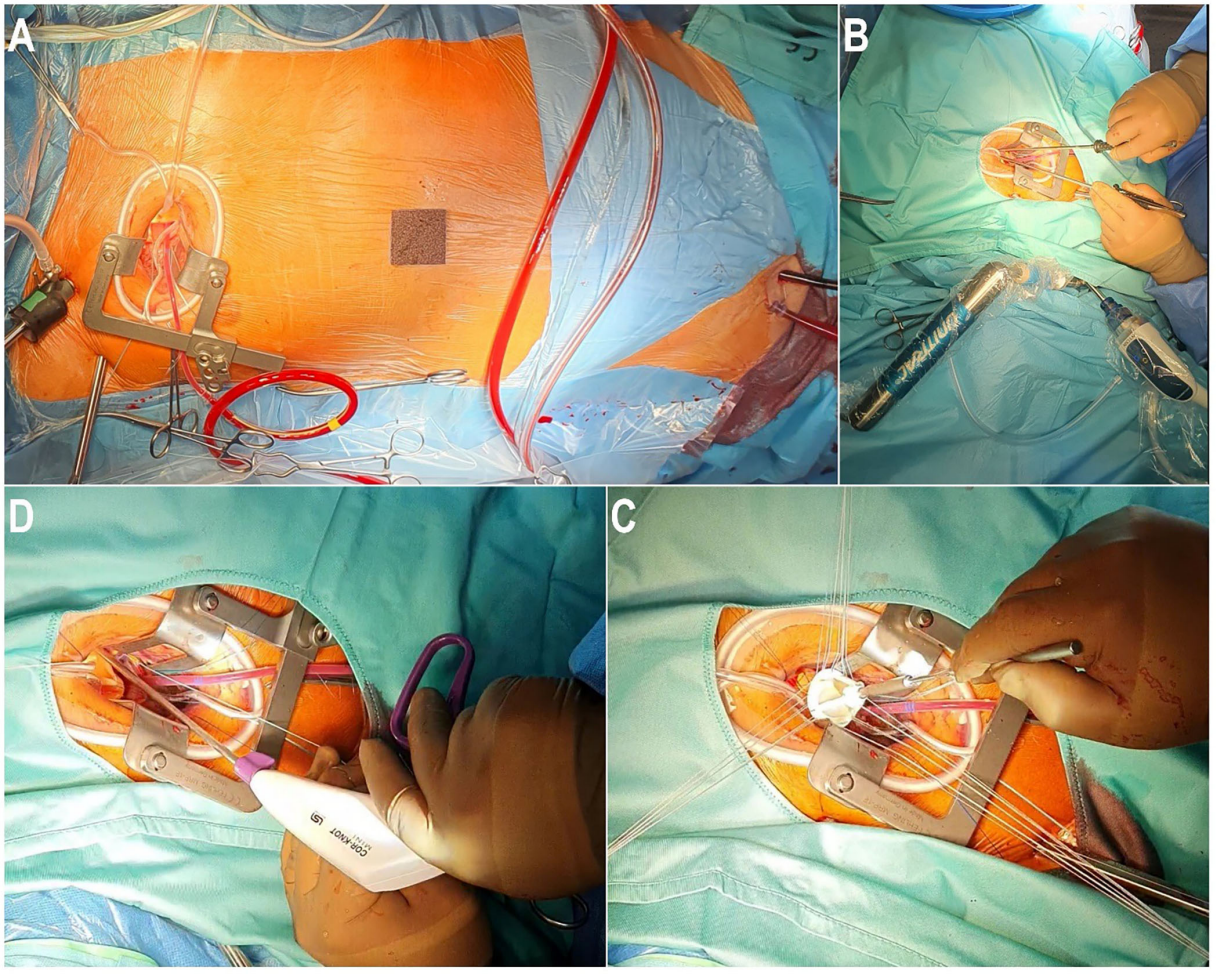
Operative setup of an endoscopic aortic valve replacement through right anterior mini-thoracotomy. **(A)** Right anterior mini-thoracotomy with percutaneous cannulation of the cardiopulmonary bypass. **(B)** Endoscopic placement of the aortic annular suture using long shaft instruments. **(C)** Implantation of the aortic valve prosthesis. **(D)** Introduction of Cor-Knot^®^ to fix the aortic valve prosthesis.

Minimally invasive cardiac surgery has been reported to be technically more challenging than conventional surgery because surgeons are confronted with a restricted view of the operating field with a concurrent, relatively long distance between the skin incision and the anatomical structures of the heart. These difficulties have been reported to be responsible for longer operating times and longer CPB and cross-clamp times observed with this procedure (8). In our view, these challenges in endoscopic MICS can be resolved and facilitated by using various devices such as a three-dimensional (3D) camera (Aesculap Einstein Vision, Tuttlingen, Germany), long surgical instruments, and an automatic suture fastener system (Cor-Knot^®^, LSI Solutions, Rochester, NY, USA) (Figure 1).

a) The use of a 3D camera is preferred:
- to enable the surgery to be undertaken in an endoscopic manner using only a soft tissue retractor without rib resection and without using a rib retractor to reduce post-operative pain,
- to securely place the Chitwood clamp (Scanlan International, Inc., St Paul, MN, USA) on the aorta under full camera visualization to avoid any possible injury to the pulmonary artery or the left atrial appendage, and
- to enable the surgeon to see all parts of the operating field and to properly resect the leaflets, to radically decalcify the valve annulus, and to precisely place the annulus sutures, especially in cases of left-sided deviated heart or the bicuspid valve with limited exposure of the aortic valve (AV).
b) Likewise, the use of long surgical instruments enables access to all structures of the aorta and the heart.
c) The fixation of the valve prosthesis in the annulus can also be more easily, rapidly, and securely performed by using automated knot technology.

All these instruments simplify and facilitate the procedure, thereby reducing the operating time without compromising the safety and the efficacity of the technique (3).

## Lesson 3: Learning curve of the surgeon

As regards the learning curve, we believe that beginner surgeons must have at least experience with performing conventional surgery involving a minimum of 100 cases in each procedure [AV, MV, and tricuspid valve (TV) surgery]. Simultaneously, they must undergo or should have undergone dry training with an endoscope, which is mandatory to achieve an imagination in endoscopic surgery. The first cases must be carefully selected for endoscopic MICS by considering the body mass index (BMI) under 30, favorable anatomical conditions of the heart and the chest, and a non-complex underlying pathology.

The assistance of an experienced surgeon in endoscopic MICS is a fundamental requirement at this learning stage. Another aspect that contributes to facilitating the surgical procedure is clear and effective communication between the surgeon, the anesthetist, the perfusionist, and the scrub nurse. Considerable experience in performing MICS is required for all the above mentioned members (3).

From our experience, we can easily speculate that new surgeons feel technically safe from 50 cases onward and the operative time decreases by increasing the number of operated cases.

## Lesson 4: Cannulation and clamping

Arterial and venous femoral cannulation for the CPB is the standard procedure used to perform an endoscopic MICS through mini-thoracotomy. Preoperative CTA of the aorta is crucial to determining whether the MICS procedure is suitable for the patient (4). Severe calcification of the femoral, abdominal, and/or thoracic aorta, and/or severe kinking in the aorta constitute contraindications for arterial femoral cannulation for CPB (Table 2). Indeed, arterial femoral cannulation for CPB in the right axillary artery represents an excellent alternative to avoid low cerebral perfusion and retrograde perfusion in cases of calcification of the abdominal, iliac, or thoracic aorta. Previous surgery of the groin, the presence of femoral or abdominal/thoracal aortic stent/prosthesis, and fungal groin infection represent further contraindications for femoral cannulation for CPB.

**Table 2:** Contraindications for arterial femoral cannulation for cardiopulmonary bypass.

**Severe calcification of the femoral or iliac artery**
Severe calcification of the abdominal and/or thoracal aorta
Severe kinking in the aorta
Femoral or abdominal/thoracal aortic stent/prosthesis
Groin infection

At the beginning of a MICS procedure, surgical access in the right groin is made through a 2- to 3-cm skin incision below the inguinal ligament, followed by insertion of the femoral cannula (Bio-Medicus multistage femoral venous cannula, Medtronic, Minneapolis, MN, USA) to the level of the superior vena cava (SVC) and the arterial cannula (Bio-Medicus arterial cannula, Medtronic, Minneapolis, MN, USA) to the level of the right external iliac artery. While in the conventional open surgical approach, the common femoral artery is directly sutured under direct vision after decannulation, the percutaneous innovative collagen-based MANTA™ Vascular Closure Device (VCD) (Essential Medical, Inc., Malvern, PA, USA) is used in MICS. It serves as an elegant, safe, and reproducible closure device to manage this small bore access by immediate sealing (9). Otherwise, suture-based vascular closure devices, such as Perclose ProGlide and ProStar XL (Abbott Vascular, Santa Clara, CA, USA), are readily used for the closure of large bore access. Notably, we recommend new surgeons in endoscopic MICS initiate the learning curve with surgical access and closure for CPB cannulation. After 50 cases, ultrasound-guided percutaneous femoral cannulation for CPB can be used in combination with the use of MANTA™ VCD to avoid surgical complications of the groin. Our experience with MANTA^TM^ system demonstrates this tool to be an effective, fast, and safe device, which also has a positive effect on the operating time compared to the surgical access for CPB in endoscopic MICS (9, 10).

As regards clamping of the aorta, we routinely use the Chitwood clamp. Alternatively, endoaortic balloon (EAB) occlusion with the endoaortic balloon clamp (Johnson & Johnson Corp, New Brunswick, NJ, USA), which is introduced from the femoral artery into the ascending aorta right above the sinotubular junction, can be used (11). Crystalloid cardioplegia (Custodiol; Koehler Chemi, Alsbach-Haenlien, Germany) is administered in an antegrade fashion through a long cardioplegia catheter (Medtronic DLP 9F, Ref 10012) into the ascending aorta and then directly into the coronary ostia in cases of aortic regurgitation.

## Lesson 5: Aortic valve

The partial upper sternotomy (PUS) procedure remains the best surgical access in MICS for AVR, which can be performed by a wide range of surgeons (4). However, endoscopic minimally invasive AV surgery *via* right anterior mini-thoracotomy (RAMT) is a safe and feasible technique without compromising on the surgical quality, the post-operative outcomes, or the patient safety when performed by a team very well-experienced in performing MICS (3, 4). Computed tomography (CT) criteria for eligibility for AVR *via* RAMT with regard to the assessment of the ascending aorta and AV position and depth are previously described in the literature (12). Severe calcified or small aortic annulus (<19 mm), hostile aortic root or ascending aorta, extensive endocarditis, severe adhesions of the lung, and/or extreme left deviated heart axis remain the common contraindications for AVR *via* RAMT. In our opinion, standard lung ventilation with a 1-lumen tube is sufficient for performing this technique (3).

The skin incision is limited to 3–5 cm longitudinally and 3 cm to the right of the midline of the sternum at the level of the third intercostal space (ICS). The chest wall access is a keyhole through the third ICS using a soft tissue retractor (ValveGate™ Soft Tissue Protector, Geister, Germany) for optimal exposure without resection or dislocation of the rip and for preserving the right internal thoracic artery and vein intact. The 3D camera port access and Chitwood clamp are usually placed medially and laterally, respectively, *via* the second ICS. The pericardium is opened 5 cm above the phrenic nerve between the innominate vein cranial and the inferior vena cava (IVC) caudal. The use of two stay sutures on the right side of the pericardium superiorly and inferiorly to the right superior pulmonary vein helps to reduce the work distance. Multiple 4–0 Prolene stay sutures in the aortic wall and the aortic valve commissures help in the exposure of the valve. In some cases, the AV annulus sits below the third ICS, requiring surgeons to face the challenge of working through a tunnel. Therefore, long-shaft instruments belong to the standard surgical setup for MICS. For a newly established surgeon in MICS, patient selection is an important step, and they should carefully consider the pre-operative anatomical CTA findings of the concerned patient (3).

The use of the 3D camera in AV surgery allows the surgeon to resect the leaflets properly, radically decalcify the valve annulus, and place the annulus sutures precisely, especially in the right coronary sinus. In cases of bicuspid AV, the role of the 3D camera is very important for localizing the left and right coronary sinus for a geometric ideal placement of the AV prosthesis beginning from the knots at the right coronary sinus (3).

## Lesson 6: Mitral valve

For the MV, endoscopic MICS has been performed extensively over the last three decades using various techniques such as the parasternal and transsternal approaches and partial upper and lower sternotomy, allowing for direct visualization and manipulation of the MV (13). Video-assisted RAMT for MV surgery remains the most commonly used MICS as it has several advantages compared to CS (14). The challenge of using this technique is to provide an equal or superior surgical outcome to conventional procedures, ensuring intraoperative quality control by documenting a successful elimination of significant mitral regurgitation (MR).

Through a 3–5 cm skin incision or a peri-areolar skin incision with a nipple-cut approach over the fourth ICS and with the optimization with assistance from a 3D camera, the exposure of the MV is obtained through dissection of the interatrial groove, left atriotomy, and using a left atrial retractor (Valve Gate™ Holders Set Mitral, Geister, Germany). Considering their excellent long-term durability, simple, and efficacious MV repair is preferred over MV replacement in the treatment of degenerative MR in terms of superior early and late survival, improved reverse ventricular remodeling and ejection fraction (EF) recovery, and a better quality of life (15, 16). MICS for MV repair provides excellent exposure of the MV and the sub-valvular apparatus, including the base of papillary muscles, allowing an optimal placement of sub-annular sutures for ring annuloplasty, leaflet resection, or augmentation, as well as uncomplicated implantation of the loops and polytetrafluorethylene neo-chords on the corresponding papillary muscle and MV leaflet when the loop technique is required (17).

The complexity of MV reconstruction makes the MICS procedure more challenging for surgeons who require a longstanding experience in this technique. Thus, we believe that surgeons must first become thoroughly proficient in performing the MICS for MV replacement and standard open MV repair before practicing these techniques in an almost closed chest.

Moreover, this technique allows the performance of concomitant procedures when cryomaze ablation, closure of a patent foramen oval, and/or left atrial appendage closure using AtriClip^TM^ (AtriCure, Inc., Mason, OH, USA) are required. In this regard, concomitant trans-mitral septal myectomy and MV surgery *via* RAMT were enrolled in our experience in endoscopic MICS performing 14 cases safely with excellent surgical outcomes (17). Moreover, the endoscopic MICS approach for MV reoperation in selected high-risk patients seems to be safe and feasible (18).

## Lesson 7: Tricuspid valve

Minimally invasive cardiac surgery for the tricuspid valve (TV) is mostly performed as a concomitant procedure to MV surgery with increased incidence due to various reasons, which include an increase in the implantation of intracardiac devices and the prevalence of atrial fibrillation (19). Venous cannulation in cases of MICS for TV varies between cardiac centers considering the expertise of the team, the cost-effectiveness of the procedure, and the reproducibility of the procedure (20). Classically, percutaneous bicaval venous cannulation through the external jugular and femoral veins and arterial cannulation through the common femoral artery is the most performed cannulation technique for CPB in MICS for TV at our department. To switch to total bypass, we use Bulldog vascular clamps for the superior vena cava and the inferior vena cava due to their effectiveness and rapid use without resorting to additional dangerous manipulations. The TV is exposured through the right atrium using an atrial retractor (ValveGate™ Holders Set Tricuspidal, Geister, Germany). Thereafter, complex surgical techniques for the repair and replacement of the TV can be safely performed in a beating heart fashion with the same quality as those of the CS approach (21, 22). Beating heart MICS techniques decrease or eliminate potential myocardial injury from ischemia time and the spare additional maneuvers of aortic cross-clamping and clamp release.

## Conclusion

Our experience with endoscopic MICS suggests that this concept can be safely, effectively, and reproducibly performed by a wide range of surgeons. This study is intended to serve as a template for newly established cardiac surgeons in minimally invasive techniques in the hope of accelerating the learning curve while improving patient outcomes.

## Data availability statement

The raw data supporting the conclusions of this article will be made available by the authors, without undue reservation.

## Author contributions

AE-S and FB contributed to conception and design of the study. SS wrote the first draft of the manuscript. AE-S wrote sections of the manuscript. All authors contributed to manuscript revision, read, and approved the submitted version.
